# A retrospective evaluation of the clinical efficacy of occlusal splint therapy combined with manual therapy in patients with anterior disc displacement without reduction

**DOI:** 10.22514/jofph.2025.055

**Published:** 2025-09-12

**Authors:** Jingke Gu, Yukang Zhang, Wanghui Ding, Shuyan Liu

**Affiliations:** ^1^Center for Plastic & Reconstructive Surgery, Department of Dental Medicine, Zhejiang Provincial People’s Hospital (Affiliated People’s Hospital, Hangzhou Medical College), 310014 Hangzhou, Zhejiang, China; ^2^Xianju Traditional Chinese Medicine Hospital, 317300 Taizhou, Zhejiang, China; ^3^Stomatology Hospital, School of Stomatology, Zhejiang University School of Medicine, Zhejiang Provincial Clinical Research Center for Oral Diseases, 310000 Hangzhou, Zhejiang, China

**Keywords:** Anterior disc displacement without reduction (ADDwoR), Temporomandibular disor-ders (TMD), Occlusal splint (CAD/CAM), Manual therapy, Maximum mouth opening (MMO), Visual analog scale (VAS), Mandibular functional impairment questionnaire (MFIQ)

## Abstract

**Background**: The study aimed to retrospectively evaluate the clinical efficacy of a computer-aided design/computer-aided manufacturing (CAD/CAM)-fabricated occlusal splint combined with manual therapy in patients diagnosed with anterior disc displacement without reduction (ADDwoR) of the temporomandibular joint (TMJ). **Methods**: The medical records of 65 adult patients with ADDwoR, treated between March 2022 and March 2023, were reviewed and allocated into three treatment groups based on the interventions they received, namely, occlusal splint therapy alone (n = 22), occlusal splint therapy combined with manual therapy (n = 22), or health education alone (n = 21). All participants received standardized health education. Clinical outcomes, including Maximum Mouth Opening (MMO), Visual Analog Scale (VAS) for pain, and the Mandibular Functional Impairment Questionnaire (MFIQ), were assessed at baseline and one and three months post-treatment by blinded evaluators. Statistical analyses were conducted using Python with Welch’s analysis of variance (ANOVA) and repeated measures ANOVA, and significance was set at *p* < 0.05. **Results**: The baseline demographic and clinical characteristics were similar among the three groups (all *p* > 0.05). At the three-month follow-up, no significant changes were observed in the health education group (*p* > 0.05). In contrast, both the occlusal splint group and the combined treatment group demonstrated significant improvements in MMO, VAS and MFIQ scores at both one and three months compared to baseline (all *p* < 0.05). Moreover, the combined treatment group showed significantly greater improvement in all measured outcomes than the splint-only group at each follow-up, with the most substantial differences observed at the three-month follow-up (all *p* < 0.05). **Conclusions**: This short-term non-randomized retrospective study suggests that combining CAD/CAM-fabricated occlusal splint therapy with manual therapy yielded superior pain relief and functional improvement compared to splint therapy alone or health education in patients with ADDwoR.

## 1. Introduction

Orofacial pain, including discomfort in the jaw and masticatory muscles, significantly affects daily activities and quality of life [1], with epidemiological data suggesting a global prevalence of temporomandibular disorders (TMD) being approximately 25% [2]. TMDs represent a common group of conditions characterized by jaw pain, restricted mandibular movement, joint sounds, and functional limitations. Among these, anterior disc displacement without reduction (ADDwoR) poses particular diagnostic and therapeutic challenges, as the displaced articular disc fails to return to its normal anatomical position, often resulting in persistent pain and functional impairment. This may extend beyond the orofacial region and manifest as headaches, sleep disturbances, and psychological distress.

The pathogenesis of ADDwoR is multifactorial, involving mechanical loading, systemic influences, occlusal discrepancies, and psychological factors such as stress and anxiety [3, 4]. Due to this complexity, a wide range of therapeutic approaches has been investigated, with contemporary treatment paradigms emphasizing conservative function-oriented strategies rather than focusing solely on repositioning the displaced disc [5, 6].

Among the conservative treatments for ADDwoR, occlusal splints are commonly employed to redistribute occlusal forces and alleviate masticatory muscle hyperactivity. The advent of computer-aided design/computer-aided manufacturing (CAD/CAM) technology has enabled the fabrication of occlusal splints with improved precision and individualized fit for enhanced therapeutic efficacy [7]. Additionally, manual therapy, which involves targeted mobilization and soft tissue manipulation of the temporomandibular joint (TMJ) and surrounding musculature, has shown beneficial effects in reducing muscular tension, alleviating pain, and improving jaw mobility [8].

However, recent network meta-analyses have highlighted the limitations of single-modality treatments. For instance, Al-Moraissi *et al*. [9] found that no single intervention consistently achieved optimal outcomes in pain reduction and improvement of mouth opening from 742 patients in 16 randomized controlled trials (RCTs). Based on this, combining occlusal splints with manual therapy has been proposed as a potentially synergistic approach that may more effectively address the multidimensional nature of TMD symptoms. This is consistent with current views that management of TMD, particularly ADDwoR, should address not only biomechanical dysfunction but also neuromuscular and psychosocial factors [10, 11].

The present study aimed to evaluate the clinical efficacy of CAD/CAM-fabricated occlusal splints combined with manual therapy in patients with ADDwoR, which was then compared with occlusal splint therapy alone and health education. Clinical outcomes were assessed in terms of changes in maximum mouth opening (MMO), pain intensity, and mandibular function. Overall, the findings could be used as a reference to inform more mechanism-based and patient-centered treatment strategies for TMD, which may guide clinicians toward interventions that could yield better improvements in patient outcomes. The null hypothesis was that there would be no statistically significant difference in clinical efficacy among the three treatment modalities, namely occlusal splint therapy, combined occlusal splint and manual therapy, and health education, in patients with ADDwoR.

## 2. Material and methods

### 2.1 Study design and ethical approval

This retrospective observational study was approved by the Human Research Ethics Committee of Zhejiang Provincial People’s Hospital (Approval No. KT2023010). The medical records of adult patients diagnosed with ADDwoR of the TMJ from March 2022 to March 2023 were reviewed, and those that had received one of the three treatment modalities, namely occlusal splint therapy fabricated via CAD/CAM, occlusal splint therapy combined with manual therapy, or health education alone, were considered. Health education was provided as a standard component across all groups.

Each patient underwent routine clinical and radiological examination of the TMJ. Detailed explanations regarding the etiology, progression, and treatment options for ADDwoR were provided to all patients, including the potential benefits and limitations of each intervention. Patients were allowed to select their preferred treatment modality and provided written informed consent before the start of therapy.

### 2.2 Sample size and statistical power

Eligible records from March 2022 to March 2023 were included based on predefined inclusion criteria. A total of 65 patients were identified and classified into three groups: occlusal splint therapy (n = 22), occlusal splints combined with manual therapy (n = 22), and health education alone (n = 21).

To assess whether the available sample size was sufficient to detect clinically meaningful differences, a *post hoc* power analysis was conducted. Based on previously published data [5, 12], a clinically significant change in MMO was defined as 6 mm with a standard deviation of 6.5 mm. Given the sample sizes of 21–22 cases per group, the calculated power to detect this difference was 83% at a significance level of α = 0.05. For the Visual Analog Scale (VAS), a minimal clinically important difference of 2.5 and a standard deviation of 2.5 yielded a power of 87% [13]. Regarding the Mandibular Functional Impairment Questionnaire (MFIQ), prior data in comparable populations [10] indicated that the available sample size provided 81% power to detect clinically meaningful changes. These results confirmed that the study sample was adequate for the intended statistical analyses.

### 2.3 Participants and group allocation

A total of 65 adult patients diagnosed with ADDwoR of the TMJ were included. All patients were evaluated and treated at our institution and the diagnosis was established according to the Diagnostic Criteria for Temporomandibular Disorders (DC/TMD) [12]. This was done by an associate professor with 14 years of clinical experience, who is also a board-certified oral and maxillofacial specialist and a member of the Temporomandibular Disorder Committee of the Zhejiang Provincial Stomatological Association, for accuracy and consistency of diagnoses for all participants. The study inclusion criteria were: (1) exhibiting persistent symptoms of ADDwoR lasting three months or longer, as defined by the DC/TMD guidelines [12]; (2) having pain intensity rated at 3.0 or higher on a 10-point VAS [5]; (3) demonstrating functional impairment, specifically an MMO ≤30 mm [13]; (4) having a diagnosis of ADDwoR radiographically confirmed by magnetic resonance imaging (MRI), which verified that the displaced disc did not return to its normal position during mandibular movement [6]; and (5) having complete medical documentation, including signed informed consent forms and evidence of completed treatment and follow-up.

Patients were excluded from the study if any of the following conditions was met: (1) previously undergone surgical interventions involving the TMJ; (2) had systemic conditions such as rheumatoid arthritis or osteoarthritis, which could influence TMJ function or pain; (3) received concurrent treatments for TMD, including ongoing pharmacological therapy or orthodontic treatment; (4) diagnosed with severe psychological disorders that could interfere with pain perception or self-reporting; (5) had a history of substance abuse, which could affect pain management and treatment compliance; and (6) presented with acute infections or inflammatory conditions of the TMJ at the time of diagnosis.

After a standardized clinical and radiological examination, a single-blinded clinician provided each patient with detailed information regarding the etiology, progression, and available treatment options for ADDwoR.

Based on the patient’s informed choice, they were allocated into (1) the Occlusal Splint Group (10 males and 12 females; mean age of 34.5 ± 7.2 years), (2) the Combined Treatment Group, which received occlusal splints fabricated via CAD/CAM in conjunction with manual therapy (9 males and 13 females; mean age of 33.8 ± 6.9 years), or (3) the Health Education Group (9 males and 12 females; mean age of 35.2 ± 7.5 years).

The baseline characteristics, including age, gender distribution, pain intensity measured using VAS and MMO, were comparable across all the three groups (all *p* > 0.05). As the study employed a patient preference-based allocation rather than a randomized, blinded design, participants were aware of the treatment modality they received—whether occlusal splint therapy, combined therapy, or health education. This lack of blinding may have introduced expectation-related bias, potentially contributing to false-positive results. However, since expectancy effects are theoretically present across all groups, this may have reduced the likelihood of differential bias between treatment and control arms. The detailed screening and allocation process is illustrated in Fig. 1.

**Fig. 1.** Patient Selection Process. VAS: Visual Analog Scale; MMO: Maximum Mouth Opening; MRI: magnetic resonance imaging; TMJ: temporomandibular joint; M: male; F: female.

### 2.4 Treatment procedures

All patients, regardless of group allocation, received comprehensive health education as a baseline intervention. This education covered several core components, including an explanation of the etiology and progression of TMD, guidance on proper dietary and chewing habits (*i.e.*, avoiding unilateral chewing, hard foods, and excessive gum chewing), recommendations for behavioral modifications, self-management strategies, and lifestyle adjustments aimed at preventing symptom exacerbation.

To ensure consistency and standardization of care, all educational content was delivered by certified oral and maxillofacial specialists using standardized materials and protocols.

Patients in the Health Education Group received only this baseline health education without any additional therapeutic interventions. They were scheduled for monthly follow-up visits to monitor their clinical condition and to reinforce self-management practices, during which, the educational content was reviewed and tailored based on each patient’s specific needs. Compliance with the recommended behavioral modifications was also assessed and documented at each visit.

Patients in the Occlusal Splint Group underwent the Kovacs Digital Occlusal Splint (KDOS) treatment protocol. Initially, an intraoral scanner (iTero, Align Technology, Santa Clara, CA, USA) was used to obtain a digital scan of the patient’s dentition and occlusal relationship, and based on the acquired data, a digital balancer was fabricated. Then, the patients were instructed to wear the balancer and perform guided jaw movements to establish a comfortable mandibular position, thereby reducing occlusal forces transmitted to the teeth and TMJ disc, facilitating balanced masticatory muscle activity and improving joint function. Once the optimal jaw position was determined, the data were imported into Exocad software (version 3.0, Exocad GmbH, Darmstadt, HE, Germany) for splint design. The final semi-anatomical occlusal splint was milled using the SELECT five-axis engraving machine (CAD/CAM milling equipment, Wieland Dental + Technik GmbH & Co. KG, Pforzheim, BW, Germany) [14, 15]. All clinical procedures were performed by board-certified oral and maxillofacial specialists with a minimum of five years of experience in digital splint therapy. Each patient was scheduled for three clinical appointments during the KDOS process, with any necessary adjustments completed within the first week of splint use. They were instructed to wear the splint for at least 12 hours daily, mainly during sleep. Follow-up evaluations were conducted monthly to assess therapeutic progress and splint adaptation [15]. A schematic flowchart of the KDOS treatment protocol is shown in Fig. 2.

**Fig. 2.** Flowchart of Kovacs Digital Occlusal Splint (KDOS) Treatment. (a) Dental scanning was performed using iTero. (b) Facebow registration was completed to capture the spatial relationship of the maxilla. (c) A digital balancer was fabricated based on the scanned data. (d) Guided mandibular movements were performed using the balancer to identify an optimal jaw position. (e) The final mandibular position was recorded using bite registration silicone and imported into Exocad software. (f) The occlusal splint was digitally designed with even posterior contact and canine guidance. (g) The splint was manufactured using a SELECT five-axis engraving machine. (h) Patients were instructed to wear the splint for at least 12 hours per day.

Patients in the Occlusal splints (fabricated via CAD/CAM) combined with manual therapy received both CAD/CAM-fabricated occlusal splints and manual therapy. Manual therapy included soft tissue mobilization and joint mobilization, targeting the musculature and structures surrounding the TMJ. Soft tissue mobilization involved the application of pressure to myofascial trigger points, with adjustments made according to the direction of muscle fibers to relieve tension in the temporalis, masseter, sternocleidomastoid, and other muscles associated with TMJ function. Joint mobilization comprised the following techniques: Initially, the therapist stabilized the patient’s head with the left hand and positioned the thumb of the right hand on the occlusal surface of the molar on the affected side. Downward pressure was then applied to exert traction on the joint, while the lower edge of the mandible was supported by the other fingers to facilitate stretching. After achieving adequate traction, the therapist used their fingers to grasp the posterior edge of the mandibular angle and gently advanced the joint forward into a slightly protruded position, with lateral movements incorporated as needed to enhance mobility. The therapist also placed their thumb on the lingual side of the last molar while making contact with the mandibular ramus and applied gentle lateral pressure to perform small-range capsular stretching.

During the treatment period, as the patients could not speak, they were instructed to raise their hands if they experienced any discomfort to prevent potential joint injury. The manual therapy was administered by licensed physical therapists with specialized training in TMJ rehabilitation [6] at the Temporomandibular Joint Rehabilitation Clinic once a week for 15 to 20 minutes, over a period of 3 months. Patient compliance with both occlusal splint usage and attendance at manual therapy sessions was closely monitored and systematically recorded throughout the treatment period.

### 2.5 Follow-up

The follow-up data were obtained from scheduled outpatient visits conducted at one-month and three-month intervals. During each visit, the patients underwent clinical evaluations, including measurement of MMO, assessment of pain intensity using the VAS, and completion of the MFIQ. Treatment adherence was also evaluated, and any adverse events related to the interventions were documented. All follow-up assessments were performed by clinicians who were blinded to group allocation to reduce bias and ensure objective outcome evaluation.

### 2.6 Evaluation indicators

Clinical outcomes were evaluated using indicators MMO, VAS for pain, and MFIQ at baseline, one month, and three months after the treatment initiation.

1. Maximum Mouth Opening (MMO):

MMO was measured using a standard ruler to determine the interincisal distance between the upper and lower central incisors while the patient opened their mouth as widely as possible without experiencing pain or discomfort. The measurements were recorded in millimeters (mm).

2. Visual Analog Scale (VAS) Pain Assessment:

Pain intensity was assessed using a 10-centimeter VAS. The patients were asked to mark a point on the scale that best represented their level of pain, with 0 indicating no pain and 10 representing the most severe pain imaginable. The data were collected by clinical evaluators blinded to the treatment group allocations to ensure objectivity and minimize bias.

3. Mandibular Function Impairment Questionnaire (MFIQ) Score:

Mandibular function was evaluated using the MFIQ, a validated instrument consisting of 16 items that assess the patient’s functional abilities and dietary performance. Each item was rated on a 5-point Likert scale, ranging from 0 (no difficulty) to 4 (extreme difficulty or inability to perform the task independently). The total score was obtained by summing the individual item scores, which represented the overall severity of mandibular functional impairment. Study patients completed the questionnaire independently, and trained clinical evaluators blinded to treatment allocation ensured standardized administration and data processing.

For the assessment of MMO, patients were instructed to open their mouths as widely as possible without experiencing pain or discomfort. The vertical distance between the incisal edges of the upper and lower central incisors was measured using a standard ruler. A measurement exceeding 35 mm was considered indicative of a return to normal mandibular joint function.

VAS scores were based on patients’ self-reported pain levels. Each patient marked their perceived pain intensity on a 10-centimeter scale, where 0 indicated no pain and 10 represented the most severe pain imaginable. Higher scores reflected greater pain intensity, providing a quantitative measure of subjective discomfort.

MFIQ scores were derived from a 16-item questionnaire designed to evaluate functional capacity and dietary limitations associated with TMD. Each item was rated on a scale from 0 to 4, with 0 indicating no difficulty and 4 indicating extreme difficulty or inability to perform the activity independently. The total score was calculated by summing all item scores and indicated the overall severity of mandibular functional impairment.

### 2.7 Statistical analysis

All statistical analyses were performed using Python with libraries such as NumPy, Pandas and SciPy for data handling and inferential testing. Custom scripts were developed to ensure reproducible data processing, including cleaning, descriptive statistics, and advanced statistical analyses. The Python code utilized in this study can be made available upon request to facilitate verification and replication.

Continuous variables are reported as mean ± standard deviation (SD). Given the presence of non-normal distributions and unequal variances among groups, Welch’s analysis of variance (ANOVA) was applied to compare differences across the three treatment groups, with statistical significance defined as *p* < 0.05. When significant intergroup differences were detected, Games-Howell *post hoc* tests were performed to accommodate unequal variances. To evaluate time-dependent changes within each group, repeated measures ANOVA or non-parametric alternatives such as the Friedman test were employed, depending on the distribution characteristics of the data. For multiple comparisons, Bonferroni or Tukey adjustments were applied to control for Type I error.

## 3. Results

This study conducted a comparative analysis of three treatment modalities—occlusal splint therapy (fabricated via CAD/CAM), occlusal splint therapy combined with manual therapy, and health education—on patients diagnosed with ADDwoR of the TMJ. The primary outcome measures included MMO, VAS scores, and MFIQ scores. All 65 enrolled patients completed assessments at baseline, one month, and three months. There were no losses to follow-up, withdrawals, or missing data throughout the study period.

Between-group comparisons were performed using Welch’s ANOVA followed by Games-Howell *post hoc* tests, selected due to the robustness of these methods in the presence of unequal variances. At baseline, all outcome measures were statistically comparable among the three groups. The smallest *p*-value (VAS score comparison between the health education and occlusal splint groups) was 0.37, with all other *p*-values exceeding 0.5. These findings confirm the comparability of groups at the outset, establishing a valid basis for subsequent analyses.

### 3.1 Analysis of time-course follow-up results

#### 3.1.1 1-month follow-up results

Visual Analog Scale (VAS): As shown in the right bar chart of Fig. 3, the combined treatment group demonstrated the lowest pain scores (1.73 ± 0.35), significantly lower than both the health education group (4.22 ± 0.64, *p* < 0.001) and the occlusal splint group (2.10 ± 0.53, *p* < 0.05). The occlusal splint group’s pain scores were also significantly lower than the health education group (*p* < 0.001).

**Fig. 3.** Effects of the three treatment modalities after one month of treatment. MMO: Maximum Mouth Opening; MFIQ: Mandibular Functional Impairment Questionnaire; VAS: Visual Analog Scale.

Mandibular Function Impairment Questionnaire (MFIQ): As shown in the center bar chart of Fig. 3, both the combined treatment group (11.16 ± 1.69) and the occlusal splint group (13.20 ± 3.19) showed significant functional improvement compared to the health education group (25.12 ± 4.50) (both *p* < 0.001). Further analysis indicated that the combined treatment group’s functional improvement was superior to the occlusal splint group (*p* < 0.01).

Maximum Mouth Opening (MMO): As shown in the left bar chart of Fig. 3, both the combined treatment group (35.10 ± 1.44 mm) and the occlusal splint group (33.39 ± 2.42 mm) exhibited significantly greater mouth opening ability than the health education group (21.21 ± 2.66 mm, *p* < 0.001). The combined treatment group also showed significantly better improvement in mouth opening compared to the occlusal splint group (*p* < 0.05).

#### 3.1.2 3-month follow-up results

Visual Analog Scale (VAS): As shown in the right bar chart of Fig. 4, with extended treatment time, the combined treatment group’s pain scores further decreased (0.93 ± 0.39), remaining significantly lower than both the health education group (4.07 ± 0.46, *p* < 0.001) and the occlusal splint group (1.88 ± 0.60, *p* < 0.01). The occlusal splint group continued to show significant pain reduction compared to the health education group (*p* < 0.001).

**Fig. 4.** Effects of the three treatment modalities after three months of treatment. MMO: Maximum Mouth Opening; MFIQ: Mandibular Functional Impairment Questionnaire; VAS: Visual Analog Scale.

Mandibular Function Impairment Questionnaire (MFIQ): As shown in the center bar chart of Fig. 4, at the 3-month follow-up, both the combined treatment group (9.30 ± 2.10) and the occlusal splint group (11.60 ± 2.40) maintained significantly lower MFIQ scores than the health education group (23.96 ± 3.74, *p* < 0.001). The combined treatment group’s functional improvement continued to exceed that of the occlusal splint group (*p* < 0.01).

Maximum Mouth Opening (MMO): As shown in the left bar chart of Fig. 4, both the combined treatment group (38.20 ± 1.78 mm) and the occlusal splint group (34.89 ± 2.63 mm) exhibited significantly greater mouth opening ability than the health education group (22.43 ± 3.60 mm, *p* < 0.001). At this time point, the combined treatment group showed a more pronounced advantage over the occlusal splint group (*p* < 0.01).

### 3.2 Comparison of each treatment over different time points

Within-group comparisons across different time points revealed no significant changes in the health education group, with MFIQ scores decreasing only slightly from 26.25 ± 1.37 at baseline to 24.40 ± 2.67 at three months, MMO increasing marginally from 20.75 ± 2.13 mm to 22.43 ± 3.51 mm, and VAS scores declining from 4.55 ± 0.46 to 4.13 ± 0.45 (all *p* > 0.05), indicating limited therapeutic benefit (Figs. 5,6,7).

**Fig. 5.** Maximum mouth opening over time for different treatments with significance. **: *p* < 0.01; ***: *p* < 0.001.

**Fig. 6.** VAS score over time for different treatments with significance. **: *p* < 0.01; ***: *p* < 0.001. VAS: Visual Analog Scale.

**Fig. 7.** MFIQ over time for different treatments with significance. *: *p* < 0.05; **: *p* < 0.01; ***: *p* < 0.001. MFIQ: Mandibular Functional Impairment Questionnaire.

Comparatively, the occlusal splint group exhibited significant clinical improvement from baseline to the one-month follow-up. Its MFIQ scores decreased from 26.15 ± 1.56 to 14.10 ± 2.91 (*p* < 0.001), MMO increased from 21.74 ± 2.19 mm to 33.14 ± 2.22 mm (*p* < 0.001), and VAS scores decreased from 4.69 ± 0.49 to 2.14 ± 0.50 (*p* < 0.001) (Figs. 5,6,7). However, between the one-month and three-month assessments, no further significant improvements were observed in MMO (33.14 ± 2.22 mm *vs*. 34.64 ± 2.39 mm, *p* > 0.05) or VAS scores (2.14 ± 0.50 *vs.* 1.86 ± 0.55, *p* > 0.05). The only continued improvement during this interval was noted in MFIQ scores, which further decreased from 14.10 ± 2.91 to 11.75 ± 2.64 (*p* < 0.05).

The combined treatment group demonstrated significant and consistent improvements across all time points. At one month, MFIQ scores were reduced from 26.40 ± 1.50 to 11.20 ± 1.94 (*p* < 0.001), MMO increased from 21.74 ± 1.78 mm to 35.05 ± 1.36 mm (*p* < 0.001), and VAS scores decreased from 4.64 ± 0.47 to 1.75 ± 0.33 (*p* < 0.001) (Figs. 5,6,7). By three months, further significant improvements were observed, with MFIQ scores decreasing to 5.00 ± 1.82 (*p* < 0.01), MMO increasing to 38.20 ± 1.68 mm (*p* < 0.01), and VAS scores decreasing to 0.93 ± 0.38 (*p* < 0.01), compared to their respective one-month values.

Overall, while both active treatment groups showed significant improvements relative to the health education group by the one-month follow-up, only the combined therapy group continued to exhibit significant and progressive improvement throughout the three months of follow-up. In contrast, the occlusal splint group showed initial improvement but plateaued after one month, while the combined treatment group achieved both immediate and progressive improvements throughout the study period.

## 4. Discussion

This study tested the null hypothesis that there would be no significant difference in clinical efficacy among the three treatment modalities. The results rejected the null hypothesis, demonstrating that the combination of occlusal splints with manual therapy significantly improves mouth opening, pain intensity, and mandibular function compared to occlusal splint therapy alone or health education.

Patients with ADDwoR of the TMJ frequently experience substantial pain, restricted mandibular movement, and functional impairment, all of which can markedly diminish their quality of life [16, 17, 18]. Current clinical guidelines advocate the use of conservative and reversible treatments as the first-line approach before considering invasive options [6, 12]. Based on these, occlusal splints and manual therapy have gained widespread clinical acceptance due to increasing evidence favoring non-invasive, functionally oriented interventions for symptom management [6, 8].

Occlusal splints represent a cornerstone of conservative treatment in ADDwoR. Conti *et al*. [19] reported that various occlusal designs achieved similar clinical outcomes, suggesting that the therapeutic benefit of splints may not depend on achieving specific occlusal relationships or condylar positions. Furthermore, Silva *et al*. [20] demonstrated that hard occlusal splints could reduce occlusal force transmission to the teeth and TMJ disc, particularly when applied to a limited dental arch segment. Numerous studies have confirmed that occlusal splints could reduce masticatory muscle-related pain, alleviate TMJ-related headaches, and improve mandibular function [17, 18, 21, 22]. In particular, Manrriquez *et al*. [18] reported that stabilization splints significantly reduced both the intensity and frequency of headaches in patients with TMD-associated comorbidities. Similarly, Emshoff *et al*. [21] and Major *et al*. [22] reported substantial improvements in muscle pain and functional capacity with splint therapy.

Despite the documented benefits of occlusal splints, conventional fabrication methods remain time-consuming, technique-sensitive, and less precise. The transition to digital workflows in dentistry has addressed many of these limitations [15, 23, 24, 25]. For instance, CAD/CAM techniques offer improved accuracy, reproducibility, and reduced procedural complexity and chairside time [15, 23, 24, 25]. Previous studies have shown that the KDOS system significantly alleviates symptoms and enhances the quality of life in patients with ADDwoR [14, 15]. The digital fabrication process enables precise and reproducible splint construction to minimize the need for frequent post-insertion adjustments [26]. In line with these findings, our results demonstrated that patients treated with CAD/CAM-fabricated occlusal splints experienced significant symptomatic improvement at both one and three months.

However, a notable pattern observed in our study, as well as in prior research, is that although occlusal splints can provide rapid initial relief, their therapeutic effects tend to plateau over time [17, 27, 28]. Conti *et al*. [17] and Wassell *et al*. [29] reported early symptomatic improvements that stabilized after several weeks of use, suggesting that additional therapeutic interventions may be necessary to sustain or further enhance clinical outcomes. Similarly, Kuzmanovic *et al*. [30] and El-Shaheed *et al*. [31] indicated that while stabilization splints were effective in the short term, their long-term efficacy, particularly in reducing pain, was limited when used alone.

In this context, manual therapy offers a complementary and potentially synergistic treatment modality. Manual therapy comprises a series of hands-on techniques aimed at improving local blood circulation, alleviating muscle tension, enhancing relaxation, and increasing joint mobility [8, 32, 33, 34]. Meta-analyses and network meta-analyses have consistently identified manual therapy as one of the most effective therapeutic options for myogenous TMD [8, 32]. For instance, Armijo-Olivo *et al*. [8] and Calixtre *et al*. [32] demonstrated that manual therapy significantly reduces pain and improves mandibular function. Furthermore, the network meta-analysis by Al-Moraissi *et al*. [33] ranked manual therapy higher than occlusal appliances in terms of its effectiveness in increasing MMO and relieving TMD-related symptoms.

In our study, combining CAD/CAM-fabricated occlusal splints with manual therapy utilized the distinct therapeutic advantages of each intervention and showed that the combined treatment group not only experienced more rapid clinical improvement by the one-month follow-up but also demonstrated continued and enhanced benefits at the three-month mark compared to the splint-only group. Occlusal splints primarily address biomechanical issues such as occlusal discrepancies and joint stabilization, while manual therapy targets neuromuscular and soft tissue dysfunctions. By addressing both occlusal and myofascial components, the combined approach produced a synergistic therapeutic effect, surpassing the efficacy of either modality alone.

Our results showed that the health education group did not exhibit substantial clinical improvement and, in fact, experienced a slight decline in mandibular function over the three-month period. Health education alone failed to produce statistically significant benefits in the short term. Although some studies have reported spontaneous remission of symptoms in patients with ADDwoR over longer observation periods (≥6 months) [ 26, 27, 33], a three-month timeframe is likely insufficient to observe such natural recovery. The discrepancy between our findings and those of long-term observational studies [27, 28] suggests that while health education is an important supportive measure, it is inadequate as a stand-alone intervention for achieving meaningful short-term clinical outcomes. Instead, it should be incorporated as an adjunctive component to active treatment modalities to guide patients toward beneficial self-care behaviors while other therapies directly address the underlying pathophysiological mechanisms.

Although the health education group showed minor subjective and objective improvements, these changes did not reach statistical significance at either the one-month or three-month follow-ups. Although previous studies suggested that some patients with ADDwoR may experience spontaneous improvement over time, particularly beyond three months [16, 17, 18], our findings indicate that within a three-month interval, health education alone is insufficient to generate significant clinical change.

In contrast, the occlusal splint group demonstrated clear short-term benefits, with significant improvements from baseline to the one-month follow-up (*p* < 0.01), although no additional significant changes were observed between the one-month and three-month timepoints (*p* > 0.05). Comparatively, the combined treatment group continued to show significant improvements across all assessed timepoints, with the magnitude of improvement between the one-month and three-month evaluations being also statistically significant (*p* < 0.01), thereby suggesting that the combined intervention not only achieved rapid symptom relief but also sustained and enhanced therapeutic outcomes over time (Figs. 5,6,7).

This study focused on adult patients diagnosed with ADDwoR, and the primary clinical objectives were to relieve symptoms and improve quality of life. Particular emphasis was placed on increasing MMO, reducing pain, and restoring mandibular function. The therapeutic goal was not to anatomically reposition the displaced disc to its original location but rather to achieve functional and symptomatic improvement. Future research could incorporate radiographic assessments to further elucidate the relationship between clinical improvement and underlying anatomical changes, as well as examine alterations in the disc-condyle relationship, joint space configuration, condylar bone morphology, and disc length following different therapeutic interventions. A deeper understanding of these structural parameters would facilitate the refinement of treatment strategies and contribute to improved outcomes for patients with TMJ disorders.

This study has several limitations worth acknowledging. First, this was a retrospective observational study with patient preference-based allocation, which introduces the potential for expectation-driven bias, as participants were aware of the treatments they selected. The primary rationale for not employing randomization was to respect individual treatment preferences, thereby enhancing patient satisfaction and adherence to prescribed protocols. While this pragmatic approach has clinical merit, it limited the ability to fully implement randomization and blinding, which may have introduced bias into outcome assessments. Although efforts were made to standardize treatment delivery and control for potential confounders, the absence of randomization remains a key methodological limitation. Future studies should incorporate RCT designs to validate these findings and reduce the risk of bias, thereby strengthening the evidence base for the combined treatment approach. Second, this study exclusively investigated symptom improvement in patients with ADDwoR using conservative management strategies. Other subtypes of TMD, which differ in etiology, pathophysiology, and response to therapy, were not examined. Given the heterogeneity of TMD, future research should stratify patients by subtype to facilitate a more precise evaluation of treatment effects. Such stratification would support the development of more individualized and subtype-specific therapeutic approaches. Third, while group sizes in this study were relatively balanced and allowed for appropriate statistical comparison, they were determined based on a clinical strategy that encouraged treatment diversification once a minimum number of patients had been enrolled in each group. Although this approach was practical in a clinical setting, it may have introduced selection bias and limited the generalizability of the findings. Future prospective RCTs with predefined group allocation and larger sample sizes are warranted to confirm the comparative effectiveness of these treatment modalities.

## 5. Conclusions

This study demonstrates that combining CAD/CAM-fabricated occlusal splints with manual therapy was significantly more effective than occlusal splint therapy alone or health education in improving MMO, reducing pain, and enhancing mandibular function in patients with ADDwoR, with both rapid symptom relief and sustained clinical improvement over a three-month period, contributing to an overall enhancement in patient quality of life. Despite the inherent limitations of its retrospective design and the potential for selection bias, this study supports a combined conservative treatment strategy for the effective management of ADDwoR. Future RCTs are needed to validate these findings and further establish the therapeutic value of integrated, non-invasive interventions in the management of TMD.

## References

[1] May A, Benoliel R, Imamura Y, Pigg M, Baad-Hansen L, Svensson P, *et al*. Orofacial pain for clinicians: a review of constant and attack-like facial pain syndromes. Cephalalgia. 2023; 43: 3331024231187160. 10.1177/03331024231187160 37548299

[2] Zieliński G, Pająk-Zielińska B, Ginszt M. A meta-analysis of the global prevalence of temporomandibular disorders. Journal of Clinical Medicine. 2024; 13: 1365. 10.3390/jcm13051365 PMC10931584 38592227

[3] Minervini G, D’Amico C, Cicciù M, Fiorillo L. Temporomandibular joint disk displacement: etiology, diagnosis, imaging, and therapeutic approaches. The Journal of Craniofacial Surgery. 2023; 34: 1115–1121. 10.1097/SCS.0000000000009103 36730822

[4] Delpachitra SN, Dimitroulis G. Osteoarthritis of the temporomandibular joint: a review of aetiology and pathogenesis. British Journal of Oral and Maxillofacial Surgery. 2022; 60: 387–396. 10.1016/j.bjoms.2021.06.017 35307273

[5] Dworkin SF, LeResche L. Research diagnostic criteria for temporomandibular disorders: review, criteria, examinations and specifications, critique. Journal of Craniomandibular Disorders: Facial & Oral Pain. 1992; 6: 301–355. 1298767

[6] Okeson JP. Management of temporomandibular disorders and occlusion. 7th edn. Elsevier Health Sciences: St. Louis, MO. 2013.

[7] Somogyi A, Végh D, Róth I, Hegedüs T, Schmidt P, Hermann P, *et al*. Therapy for temporomandibular disorders: 3D-printed splints from planning to evaluation. Dentistry Journal. 2023; 11: 126. 10.3390/dj11050126 PMC10217112 37232777

[8] Armijo-Olivo S, Pitance L, Singh V, Neto F, Thie N, Michelotti A. Effectiveness of manual therapy and therapeutic exercise for temporomandibular disorders: systematic review and meta-analysis. Physical Therapy. 2016; 96: 9–25. 10.2522/ptj.20140548 PMC4706597 26294683

[9] Al-Moraissi EA, Al-Otaibi K, Almaweri AA, Bastos RM, Haas Junior OL, Amran AG. Treatment of painful temporomandibular joint disc displacement without reduction: network meta-analysis of randomized clinical trials. International Journal of Oral and Maxillofacial Surgery. 2024; 53: 584–595. 10.1016/j.ijom.2024.02.004 38395688

[10] Kapos FP, Exposto FG, Oyarzo JF, Durham J. Temporomandibular disorders: a review of current concepts in aetiology, diagnosis and management. Oral Surgery. 2020; 13: 321–334. 10.1111/ors.12473 PMC8631581 34853604

[11] LeResche L. Epidemiology of temporomandibular disorders: implications for the investigation of etiologic factors. Critical Reviews in Oral Biology & Medicine. 1997; 8: 291–305. 10.1177/10454411970080030401 9260045

[12] Schiffman E, Ohrbach R, Truelove E, Look J, Anderson G, Goulet JP, *et al*.; International RDC/TMD Consortium Network, International association for Dental Research; Orofacial Pain Special Interest Group, International Association for the Study of Pain. Diagnostic criteria for temporomandibular disorders (DC/TMD) for clinical and research applications: recommendations of the international RDC/TMD Consortium Network* and Orofacial Pain Special Interest Group^†^. Journal of Oral & Facial Pain and Headache. 2014; 28: 6–27. 10.11607/jop.1151 PMC4478082 24482784

[13] Manfredini D, Guarda-Nardini L, Winocur E, Piccotti F, Ahlberg J, Lobbezoo F. Research diagnostic criteria for temporomandibular disorders: a systematic review of axis I epidemiologic findings. Oral Surgery, Oral Medicine, Oral Pathology, Oral Radiology, and Endodontics. 2011; 112: 453–462. 10.1016/j.tripleo.2011.04.021 21835653

[14] Hua J, Fan X, Nie X, He D. Preliminary evaluation of Kovacs digital occlusal splint in the treatment of temporomandibular disorders: a single-centre, cross-sectional study. Journal of Oral Rehabilitation. 2023; 50: 687–697. 10.1111/joor.13466 37067077

[15] Xu Q, Li J, Wang C, Hu SQ, Chen Y, Nie X, *et al*. Evaluation of the efficacy and quality of life in patients with temporomandibular joint disorders treated with Kovacs digital occlusal splint: a pilot study. BMC Oral Health. 2024; 24: 802. 10.1186/s12903-024-04572-4 PMC11251381 39014426

[16] Sanchla AD, Shrivastav S, Kamble RH, Nerurkar SA, Toshniwal N. Altered quality of life in patients with temporomandibular joint disorders: a review. Journal of Clinical and Diagnostic Research. 2023; 17: ZE08–ZE13.

[17] Conti PC, de Alencar EN, da Mota Corrêa AS, Lauris JR, Porporatti AL, Costa YM. Behavioural changes and occlusal splints are effective in the management of masticatory myofascial pain: a short-term evaluation. Journal of Oral Rehabilitation. 2012; 39: 754–760. 10.1111/j.1365-2842.2012.02327.x 22672361

[18] Manrriquez SL, Robles K, Pareek K, Besharati A, Enciso R. Reduction of headache intensity and frequency with maxillary stabilization splint therapy in patients with temporomandibular disorders-headache comorbidity: a systematic review and meta-analysis. Journal of Dental Anesthesia and Pain Medicine. 2021; 21: 183–205. 10.17245/jdapm.2021.21.3.183 PMC8187022 34136641

[19] Conti PC, dos Santos CN, Kogawa EM, de Castro Ferreira Conti AC, de Araujo Cdos R. The treatment of painful temporomandibular joint clicking with oral splints: a randomized clinical trial. The Journal of the American Dental Association. 2006; 137: 1108–1114. 10.14219/jada.archive.2006.0349 16873326

[20] Silva CAGD, Grossi ML, Araldi JC, Corso LL. Can hard and/or soft occlusal splints reduce the bite force transmitted to the teeth and temporomandibular joint discs? A finite element method analysis. CRANIO®. 2023; 41: 298–305. 10.1080/08869634.2020.1853464 33280545

[21] Emshoff R. Clinical factors affecting the outcome of occlusal splint therapy of temporomandibular joint disorders. Journal of Oral Rehabilitation. 2006; 33: 393–401. 10.1111/j.1365-2842.2005.01584.x 16671984

[22] Major PW, Nebbe B. Use and effectiveness of splint appliance therapy: review of literature. CRANIO®. 1997; 15: 159–166. 10.1080/08869634.1997.11746007 9586519

[23] Abad-Coronel C, Ruano Espinosa C, Ordóñez Palacios S, Paltán CA, Fajardo JI. Comparative analysis between conventional acrylic, CAD/CAM milled, and 3D CAD/CAM printed occlusal splints. Materials. 2023; 16: 6269. 10.3390/ma16186269 PMC10532716 37763547

[24] Sun X, Feng Y, Jiao Y, Liu W. Fully digital workflow for the fabrication of occlusal stabilization splints based on individual mandibular movement. Journal of Dentistry. 2024; 141: 104826. 10.1016/j.jdent.2023.104826 38157975

[25] Rabel K, Lüchtenborg J, Linke M, Burkhardt F, Roesner AJ, Nold J, *et al*. 3D printed versus milled stabilization splints for the management of bruxism and temporomandibular disorders: study protocol for a randomized prospective single-blinded crossover trial. Trials. 2024; 25: 589. 10.1186/s13063-024-08437-7 PMC11376033 39238023

[26] Muresanu SA, Hedesiu M, Dinu C, Roman R, Almasan O. Digital occlusal splints for temporomandibular joint disorders: a systematic review. Romanian Journal of Stomatology. 2022; 68: 97–105.

[27] Fang Z, Yao Y, Fan S, Jin L, Yang Y, Liu S. Physical therapy and non-surgical manual disc reduction combined with anterior repositioning splint for acute disc displacement without reduction of the temporomandibular joint in adolescents. Clinical Oral Investigations. 2024; 28: 517. 10.1007/s00784-024-05910-0 39243315

[28] Kurita K, Westesson PL, Yuasa H, Toyama M, Machida J, Ogi N. Natural course of untreated symptomatic temporomandibular joint disc displacement without reduction. Journal of Dental Research. 1198; 77: 361–365. 10.1177/00220345980770020401 9465168

[29] Wassell RW, Adams N, Kelly PJ. Treatment of temporomandibular disorders by stabilising splints in general dental practice: results after initial treatment. British Dental Journal. 2004; 197: 35–41. 10.1038/sj.bdj.4811420 15243608

[30] Kuzmanovic Pficer J, Dodic S, Lazic V, Trajkovic G, Milic N, Milicic B. Occlusal stabilization splint for patients with temporomandibular disorders: meta-analysis of short and long term effects. PLOS ONE. 2017; 12: e0171296. 10.1371/journal.pone.0171296 PMC5293221 28166255

[31] El-Shaheed NH, Mostafa AZH, Aboelez MA. Efficacy of stabilisation splint and low-level laser therapy for patients with chronic closed lock from non-reducible displaced temporo-mandibular joint discs: a parallel randomised clinical trial. Journal of Oral Rehabilitation. 2023; 50: 177–193. 10.1111/joor.13405 36564950

[32] Calixtre LB, Moreira RF, Franchini GH, Alburquerque-Sendín F, Oliveira AB. Manual therapy for the management of pain and limited range of motion in subjects with signs and symptoms of temporomandibular disorder: a systematic review of randomised controlled trials. Journal of Oral Rehabilitation. 2015; 42: 847–861. 10.1111/joor.12321 26059857

[33] Al-Moraissi EA, Conti PCR, Alyahya A, Alkebsi K, Elsharkawy A, Christidis N. The hierarchy of different treatments for myogenous temporomandibular disorders: a systematic review and network meta-analysis of randomized clinical trials. Oral and Maxillofacial Surgery. 2022; 26: 519–533. 10.1007/s10006-021-01009-y 34674093

[34] Lundh H, Westesson PL, Eriksson L, Brooks SL. Temporomandibular joint disk displacement without reduction. Treatment with flat occlusal splint versus no treatment. Oral Surgery, Oral Medicine, and Oral Pathology. 1992; 73: 655–658. 10.1016/0030-4220(92)90003-9 1437030

